# Undrained capacity of circular shallow foundations under combined VHMT loading

**DOI:** 10.1177/0309524X221083024

**Published:** 2022-03-11

**Authors:** Pengpeng He, Tim Newson

**Affiliations:** Department of Civil & Environmental Engineering, Western University, London, ON, Canada

**Keywords:** Wind turbine, circular foundation, zero-tension interface, failure envelope, finite element analysis

## Abstract

Wind turbine (WT) foundations are generally subjected to large combined vertical, horizontal, moment, and torsional (VHMT) loads. The available investigations of the ultimate limit states of WT foundations focus predominately on unlimited-tension soil-foundation interfaces that are more appropriate for offshore settings. However, the interfaces for onshore WT foundations are generally unable to resist tensile loads. To address this omission, a zero-tension interface is used to investigate the VHMT failure envelope of circular shallow foundations under undrained soil conditions using finite element analysis. The effects of soil strength heterogeneity and foundation embedment were investigated. The results show that torsional loads reduce the VHM bearing capacity of circular foundations. The foundation embedment is also found to significantly increase the foundation bearing capacity. A full 4-D analytical expression for the VHMT failure envelope has been proposed based on the calculated failure envelopes.

## Introduction

Shallow foundations have been extensively used to support large onshore and offshore wind turbines (WTs). The load-bearing capacity of WT shallow foundations under combined loads is particularly important due to the complex environmental effects. For example, the horizontal loads on a WT caused by combined winds, waves and currents are substantial, and a large tower height leads to significant moment loading on the foundation. Traditional analytical methods for these types of structure are based on classical solutions for the uniaxial vertical bearing capacity of shallow foundations. To account for the effect of load inclination and eccentricity, the load inclination factor and the effective foundation area (based on the effective width) are introduced to the conventional method, as recommended by some geotechnical design guidelines (e.g. DNV GL, 2016). However, these simple, traditional methods may not be accurate enough in some cases, because the load inclination and eccentricity effects are separately considered (Gourvenec, 2007). In general, this approach is conservative for combined *VHM* loadings (Taiebat and Carter, 2002), while it has been shown to be non-conservative for strip foundations on soils with shear strength increasing with depth (Ukritchon et al., 1998).

A more recent design approach is the failure envelope method, which explicitly incorporates the load interaction effects of various loading components (Shen et al., 2017). This method has been recommended as an alternative to conventional theory in API (2011) and ISO (2016). Failure envelopes under undrained soil conditions for different foundation geometries, soil strength profiles, and interface conditions have been considered previously (e.g. strip (Ganesh and Kumar, 2021), rectangular (Gourvenec and Randolph, 2003), circular (Suryasentana et al., 2020; Wang et al., 2020) and bucket (He et al., 2021) foundations); homogeneous strength (Taiebat and Carter, 2002), non-homogeneous strength (Feng et al., 2014; Ganesh and Kumar, 2021) and two-layer strength (Xia et al., 2021) soils; and zero-tension interface (Pham et al., 2020; Shen et al., 2016) and unlimited-tension interface (Gourvenec and Randolph, 2003; Wang et al., 2020)). These studies focused primarily on load combinations of vertical (*V*), horizontal (*H*), and moment (*M*) loads. However, environmental loads on a WT structure are often not co-planar, and transverse loads also induce torsional effects on the foundation (Bienen et al., 2007). Thus, the influence of torsional loads should not be ignored for the failure envelope of shallow foundations. Although the effects of torsional loads have been investigated by some workers previously (e.g. Abyaneh et al., 2015; Feng et al., 2017), those studies are limited to rectangular foundations using an unlimited-tension interface. This form of interface is often assumed for offshore structures, particularly for skirted foundations. However, the reliability of under-base suction in offshore environments can be conditional (Shen et al., 2016). Moreover, onshore shallow foundations can uplift and separate from the soil under large overturning moments, because the soil-foundation interface is unable to resist tensile loads. Since many of the aforementioned studies have concentrated on offshore cases with unlimited-tension interfaces, this interface condition has been generally ignored.

For onshore design practice, the existing 2-D and 3-D failure envelopes are not able to be directly applied, since an analytical equation of the general 4-D VHMT failure envelope is required. This paper aims to address two omissions in the literature: (1) the effects of torsional loads on the 4-D VHMT failure envelope for circular foundations with a zero-tension interface; and (2) the provision of an analytical 4-D VHMT failure envelope that can be directly applied to current design practice to perform simple design checks. This paper considers circular foundations with a zero-tension interface under undrained soil conditions, and the effects of soil strength heterogeneity and foundation embedment on the failure envelope.

## Finite element analysis

### Material models and interface conditions

**Figure 1.**
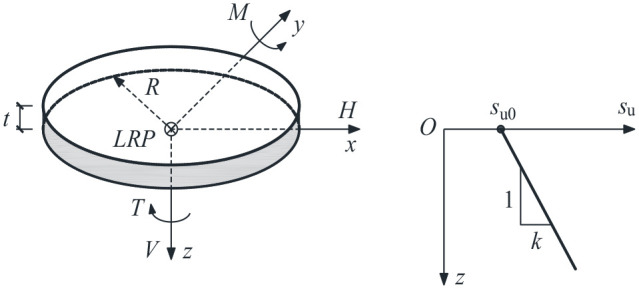
Sign conventions and soil strength profile.

A linear elastic perfectly plastic constitutive relationship with a Mohr-Coulomb (M-C) failure criterion was used to model the soil behavior. The M-C criterion devolves to the Tresca criterion under undrained conditions, which is defined by three parameters: undrained Young’s modulus, *E*_u_, Poisson’s ratio, *µ*, and undrained shear strength, *s*_u_. To study the effect of soil strength heterogeneity, the undrained shear strength was considered to linearly increase with depth from the ground surface (see Figure 1):

(1) $$s_{u}=s_{u0}+\mathrm{kz}$$

where *s*_u0_ is the undrained shear strength at foundation level; *k* is the strength increase per unit depth. In this paper, *s*_u0_ was held constant at 100 kPa and the Poisson’s ratio was taken as 0.495 (representative of an undrained condition). The dimensionless soil strength heterogeneity ratio defined by *κ *= *kD*/*s*_u0_ (Gourvenec and Randolph, 2003) was taken as 0 (homogeneous), 2, 6, and 10. A sufficiently large *E*_u_/*s*_u0_ ratio of 10,000 was selected to minimize mesh distortion (Abyaneh et al., 2015). The foundation was assumed to act as a rigid body. A load reference point (LRP) attached to the center of the foundation lower face was utilized to apply prescribed displacements or loads, as shown in Figure 1.

Similar to the approach of Shen et al. (2016), the FE analyses considered a zero-tension rough base that allows separation of the foundation from the soil. A zero-tension rough base can be modeled using a Coulomb friction condition with a friction coefficient of 20 (Shen et al., 2016). For embedded foundations, a reduced interface shear strength for the side and top interfaces is always recommended due to installation or in-service loading processes (Gourvenec and Mana, 2011). In this analysis, smooth side and top conditions (i.e. an interface adhesion factor *α *= 0 and the shear strength on the interface α*s*_u_ = 0) for the embedded foundations were considered to provide more conservative estimations. The same consideration was also made by Gourvenec and Mana (2011).

### Geometry and mesh

The FE analysis was conducted using the software ABAQUS (Dassault Systèmes, 2016). The diameter (*D*) and thickness (*t*) of the foundation were 19 and 3 m, representing typical dimensions for current onshore WTs used in North America. The embedment depth ratio, *d*/*D* (*d* is the foundation embedment depth), was taken as 0, 0.16, 0.30, and 0.50 to span cases of practical interest. To avoid the effects of model boundaries on the development of failure mechanisms, the mesh length, *L*, and mesh height, *H*, were taken as 120 and 50 m, following the recommendations of Deshpande (2016).

**Figure 2.**
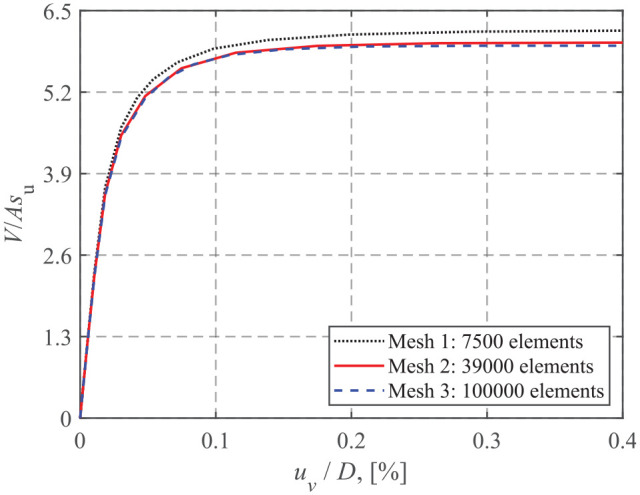
Mesh convergence study for a homogeneous soil.

**Figure 3.**
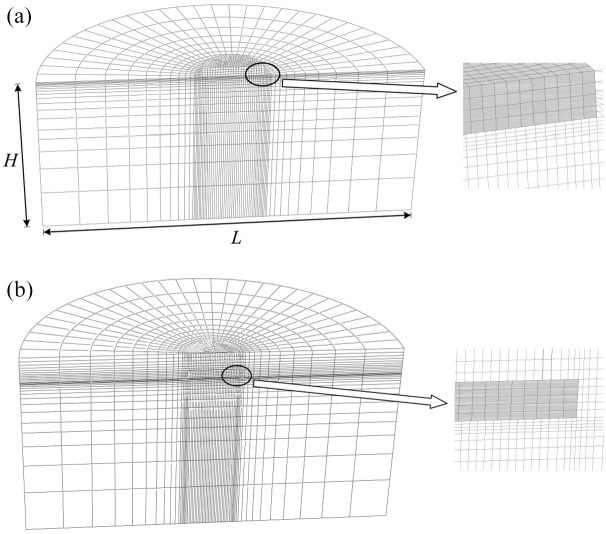
Half-view of the FE mesh: (a) surface foundation and (b) embedded foundation (*d*/*D *= 0.50).

A mesh convergence study was carried out for a number of cases, and a typical result is shown in Figure 2. The difference between the ultimate vertical loads using Mesh 2 (39,000 elements) and 3 (100,000 elements) is about 2%. However, the model solution with Mesh 3 takes about 6 times longer during processing than that using Mesh 2. Therefore, Mesh 2 was adopted in the analysis. Figure 3 shows the three-dimensional half model using Mesh 2. The mesh was composed of around 39,000 brick elements (i.e. first-order, 8-noded brick element with reduced integration and hourglass control). To capture the intense stress concentration close to the foundation edge and the large plastic shear strains at the interface, the soil regions in the vicinity of the foundation edge and the horizontal thin soil layer close to the interface were carefully refined (Gourvenec and Randolph, 2003). The cylindrical circumference of the soil was constrained to prevent out-of-plane translations, and the bottom of the soil domain was fixed in the three orthogonal directions.

### Sign conventions and loading paths

The sign conventions for the loads are also shown in Figure 1. The horizontal and moment loads were considered to be in the same plane.

Probe and swipe tests were employed to detect the failure envelopes under various load conditions. In a probe analysis, a fixed-ratio displacement is imposed on the foundation to track the failure point on the failure envelope. A probe test can only obtain a single point on an envelope.

**Figure 4.**
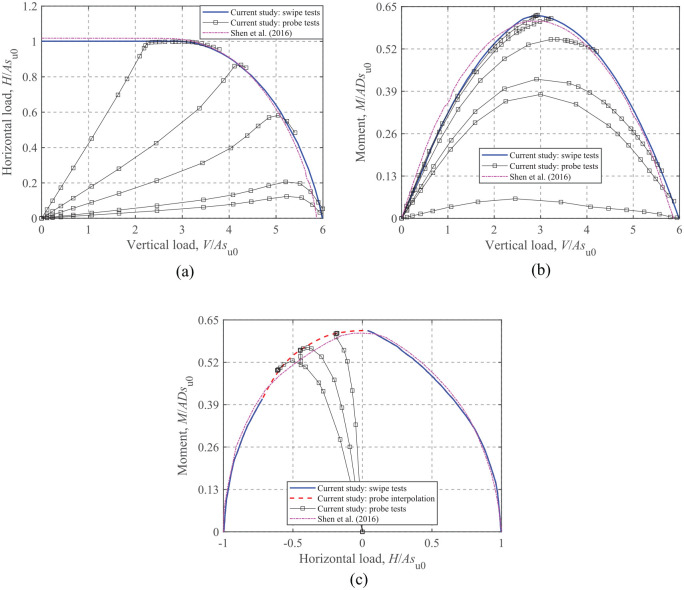
VHM failure envelopes of a surface foundation for κ = 0: (a) *H*-*V*, (b) *M*-*V*, and (c) *M*-*H* at *V*/*V*_ult_ = 0.50.

The swipe test brings the foundation to a collapse state in coordinate direction 1 first (displacement-controlled), followed by a displacement applied in coordinate direction 2, during which the increment of the displacement in coordinate direction 1 remains zero. For some cases the swipe test cannot capture the entire failure envelope due to convergence issues, hence additional probe tests were carried out to facilitate the analysis. Three typical failure envelopes obtained using both swipe and probe tests are shown in Figure 4. However, swipe tests may considerably underestimate the failure envelopes of embedded foundations (Gourvenec and Randolph, 2003). Therefore, swipe tests were performed only for the surface foundations and probe tests were utilized for the embedded foundations.

## Surface foundations on non-homogeneous soils

For VHM loading, Shen et al. (2016) has studied the failure envelopes for circular *surface* foundations on non-homogeneous soils with a zero-tension interface using numerical analysis. These envelope shapes have been confirmed during the current study and for reasons of brevity only a few cases are shown in Figure 4. There is an excellent match between the two sets of envelopes, which also provides validation of the methods used herein. In this section, the effects of torsion on the more general VHMT failure envelopes are assessed and discussed. The FE results and the corresponding closed-form equations for torsion-vertical (*T-V*), horizontal-torsion (*H-T*), and moment-torsion (*M-T*) envelopes are presented.

### Pure uniaxial capacity

The ultimate loads for vertical, horizontal and torsional modes are referred to as the corresponding uniaxial load-carrying capacities in the absence of other loading modes. As a foundation with a zero-tension interface cannot resist moments without vertical loads, the ultimate moment capacity is referred to as the maximum moment load under vertical loads (Shen et al., 2016). The uniaxial bearing capacity factors are defined as:

(2) $$\begin{array}{c} v_{0}=V_{\mathrm{ult}}/\left(As_{u0}\right)\\ \begin{array}{c} h_{0}=H_{\mathrm{ult}}/\left(As_{u0}\right)\\ m_{0}=M_{\mathrm{ult}}/\left(\mathrm{AD}s_{u0}\right) \end{array}\\ t_{0}=T_{\mathrm{ult}}/\left(\mathrm{AD}s_{u0}\right) \end{array}$$

where *A* is the soil-foundation contact area.

The estimated $v_{0}$ , $h_{0}$ , $m_{0}$ , and $t_{0}$ for different heterogeneity ratios are summarized in Table 1.

**Table 1.** Uniaxial bearing capacity factors for soils with different heterogeneity ratios.

κ	$v_{0}$	$h_{0}$	$m_{0}$	$t_{0}$
0	6.00	1.00	0.62	0.33
2	7.51	1.00	0.74	0.33
6	9.59	0.99	0.91	0.33
10	11.29	1.00	1.03	0.34

The values of $v_{0}$ , $h_{0}$ , and $m_{0}$ in Table 1 generally agree with the results of Shen et al. (2016) (with difference less than 3%). Similar to the horizontal bearing capacity, the torsional bearing capacity exhibits independence on the heterogeneity ratio (He and Newson, 2020), since the *H-T* failure state is reached only when the shear stress of $s_{u0}$ is fully developed.

### Torsion-Vertical loading

**Figure 5.**
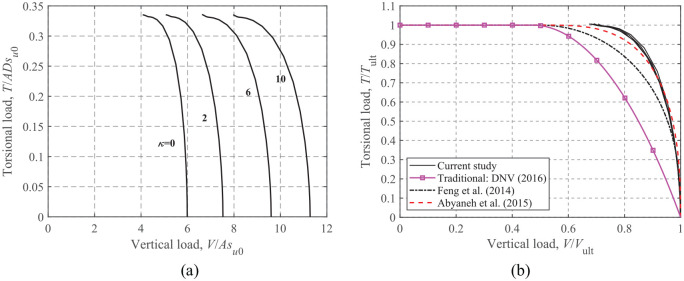
*T*-*V* failure envelopes: (a) dimensionless and (b) normalized.

The *T-V* failure envelopes for different soil heterogeneity ratios are shown in Figure 5. Figure 5(b) indicates that the *T-V* envelopes normalized by the corresponding ultimate capacities collapse into a narrow band regardless of the heterogeneity ratios.

Feng et al. (2014) provided an expression for rectangular foundations with an unlimited-tension interface, which was then applied to a zero-tension interface by Shen et al. (2017):

(3) $$\begin{array}{c} T/T_{\mathrm{ult}}={\left[1-4{\left(V/V_{\mathrm{ult}}-0.5\right)}^{2}\right]}^{0.4},\;V/V_{\mathrm{ult}}>0.5\\ T/T_{\mathrm{ult}}=1,\;V/V_{\mathrm{ult}}\leq 0.5 \end{array}$$

Abyaneh et al. (2015) proposed a similar equation for circular foundations with an unlimited-tension interface:

(4) $$\begin{array}{c} V/V_{\mathrm{ult}}=0.5+0.5{\left[1-{\left(T/T_{\mathrm{ult}}\right)}^{2.5}\right]}^{0.3},\;V/V_{\mathrm{ult}}>0.5\\ T/T_{\mathrm{ult}}=1,\;V/V_{\mathrm{ult}}\leq 0.5 \end{array}$$

Equations (3) and (4) are shown in Figure 5 for comparison. The figure shows that equation (4) provides a reasonable approximation, although it was developed for an unlimited-tension interface. In contrast, equation (3) is more conservative compared to the FE results. The traditional method (DNV GL, 2016) using the concept of the effective foundation area is also compared. Figure 5(b) shows that the DNV-derived *T-V* failure envelope has the same shape as the *H-V* envelope shown in Figure 4(a), since the torsional load in DNV GL (2016) is accounted for in the bearing capacity equation indirectly by an equivalent horizontal load, $H^{\prime }$ , that is, under the condition of *H *= 0 and *M *= 0, $H^{\prime }=4T/\left(\sqrt{\pi }R\right)$ . Compared to the FE results in Figure 5(b), the DNV-derived envelope decreases more gradually with increasing *V* and lies entirely inside the FE-calculated failure envelopes, indicating that the design practice recommended by DNV GL (2016) is probably conservative.

### Horizontal-Torsion loading

**Figure 6.**
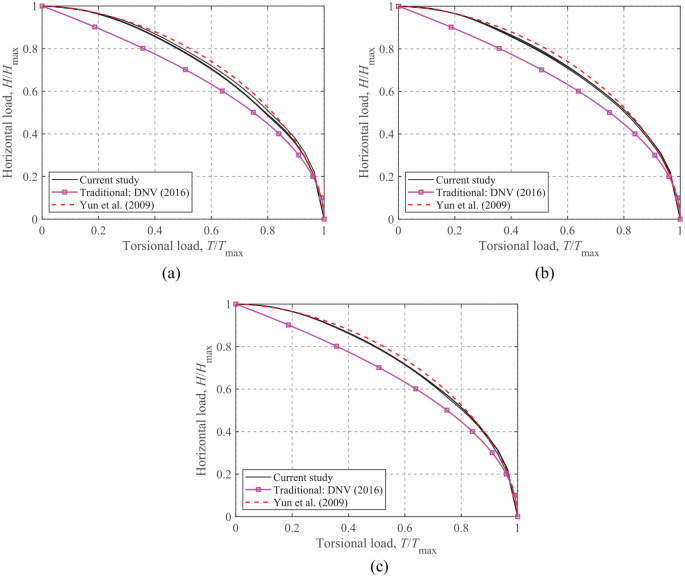
*H*-*T* failure envelopes: (a) *V*/*V*_ult_ = 0.75, (b) *V*/*V*_ult_ = 0.50, and (c) *V*/*V*_ult_ = 0.25.

Figure 6 presents the *H*-*T* envelopes normalized by the corresponding maximum values for *V*/*V*_ult_ = 0.25, 0.50, and 0.75. It can be seen that the *H*-*T* envelopes are independent of all of the soil heterogeneity ratios, since the failure mechanism under horizontal and torsional loads involves only the interface strength. Finnie and Morgan (2004) proposed equation (5) to model the *H*-*T* relationship:

(5) $${\left(H/H_{\max}\right)}^{l}+{\left(T/T_{\max}\right)}^{n}=1$$

where $H_{\max}$ and $T_{\max}$ are the maximum horizontal and torsional loads for a given $V/V_{\mathrm{ult}}$ .

As shown by equation (3), for $V/V_{\mathrm{ult}}\leq 0.5$ , $H_{\max}=H_{\mathrm{ult}}$ , and $T_{\max}=T_{\mathrm{ult}}$ . For $V/V_{\mathrm{ult}}>0.5$ , $T_{\max}$ is calculated from equation (3) as:

(6) $$T_{\max}={\left[1-{\left(2V/V_{\mathrm{ult}}-1\right)}^{3.33}\right]}^{0.4}\cdot T_{\mathrm{ult}}$$

$H_{\max}$ for $V/V_{\mathrm{ult}}>0.5$ is evaluated using Green’s original solution (Green, 1954) as $H_{\max}=[1-{\left(2V/V_{\mathrm{ult}}-1\right)}^{2}]\cdot H_{\mathrm{ult}}$ .

The dimensionless powers, *l* and *n* in equation (5), are dependent upon the foundation geometry. Yun et al. (2009) recommended *l *= *n = *1.75 for circular and square foundations with an unlimited-tension interface.

The curves for the traditional methods (DNV GL, 2016) and equation (5) with *l *= *n = *1.75 are also presented together with the FE results in Figure 6. This shows that equation (5) with *l *= *n = *1.75 can be an acceptable choice to fit the *H*-*T* failure envelopes, although it provides smaller results compared to the current study at *V*/*V*_ult_ = 0.75. In contrast, for all vertical load mobilizations, the traditional method results in more conservative failure envelopes.

### Moment-Torsion loading

**Figure 7.**
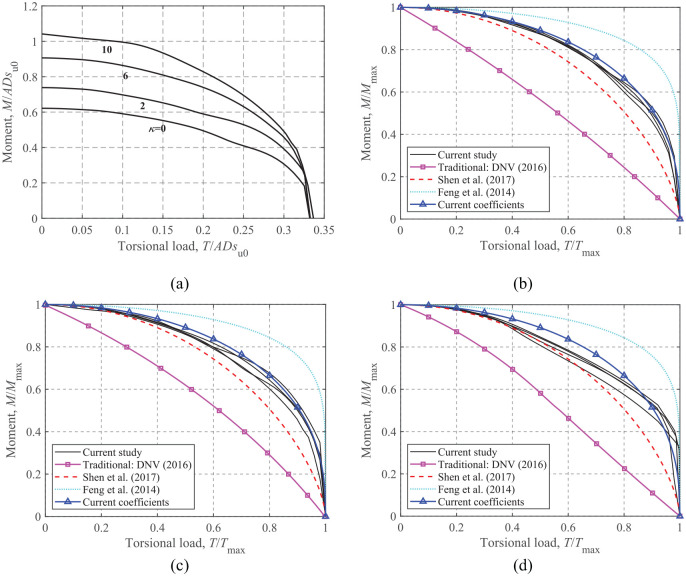
*M*-*T* failure envelopes: (a) dimensionless, *V*/*V*_ult_ = 0.50, (b) normalized, *V*/*V*_ult_ = 0.75, (c) normalized, *V*/*V*_ult_ = 0.50, and(d) normalized, *V*/*V*_ult_ = 0.25.

The ultimate load-carrying capacity under combined moment and torsional loads at *V*/*V*_ult _= 0.25, 0.50, and 0.75 for the four soil heterogeneity ratios is compared in Figure 7. The dimensionless envelopes in Figure 7(a) show the expansion of the curves with the soil heterogeneity ratio. Figure 7(b) to (d) show that the *M*-*T* envelopes normalized by the corresponding maximum loads fall into a tight band for all levels of vertical load mobilizations, which eliminates their dependence on the soil heterogeneity ratio. The results of Feng et al. (2014) and Shen et al. (2017) are also incorporated for comparison.

Figure 7 shows that the results of Feng et al. (2014) differ considerably from the current results due to two factors: (1) an unlimited-tension interface with no detachment allowed was used by Feng et al. (2014); and (2) rectangular foundations were considered by Feng et al. (2014). The figure also shows that the results of Shen et al. (2017) derived for rectangular foundations with a zero-tension interface compare better with the current FE results, since the approach of Shen et al. (2017) has the same zero-tension interface condition. This may also indicate that the interface condition exhibits a greater influence on the *M-T* envelope than the foundation shape.

Shen et al. (2017) proposed a *M*-*T* relationship for rectangular foundations under zero-tension interface conditions:

(7) $${\left(M/M_{\max}\right)}^{p}+{\left(T/T_{\max}\right)}^{q}=1,\;\mathrm{for}\;V/V_{\mathrm{ult}}\leq 0.50\leq \kappa \leq 10$$

where p and q are dimensionless parameters. Shen et al. (2017) suggested p=1.5 and q=2.0 . Based on the results for rectangular foundations with an unlimited-tension interface, Feng et al. (2014) suggested p=6.0 and q=2.0 .

The calculation of $T_{\max}$ in equation (7) also follows equation (6), and $M_{\max}$ in equation (7) for different vertical load mobilizations is evaluated based on the *M*-*V* relationship proposed by Gourvenec (2007):

(8) $$M_{\max}/M_{\mathrm{ult}}=4\left[V/V_{\mathrm{ult}}-{\left(V/V_{\mathrm{ult}}\right)}^{2}\right]$$

The analytical relationship (i.e. equation (7)) and the results from the conventional method are also shown in Figure 7. It can be seen that the curves produced with p=1.5 and q=2.0 always lie inside the envelopes, while the case of p=6.0 and q=2.0 predicts envelopes that extend significantly beyond the FE results. To gain better predictions of the current study, p and q of equation (7) can be adjusted. As shown in Figure 7, p=2.5 and q=2.0 provide more satisfactory predictions. In contrast, the envelopes derived by DNV GL (2016) yield approximately linear relationships between $T/T_{\mathrm{ult}}$ and $M/M_{\mathrm{ult}}$ and lie entirely inside the FE-calculated envelopes.

## Embedded foundations in a homogeneous soil

### Pure uniaxial capacity

The depth correction factor, $d_{c}$ , is defined as the ratio of the dimensionless capacity for embedded foundations (i.e. d/D>0 ) compared to that of surface foundations (i.e. d/D=0 ), that is,

(9) $$d_{c}=\frac{N_{d/D}}{N_{d/D=0}}$$

where $N_{d/D}=V_{\mathrm{ult}}/\left(As_{u}\right)$ is the vertical capacity; $N_{d/D}=H_{\mathrm{ult}}/\left(As_{u}\right)$ is the horizontal capacity; and $N_{d/D}=M_{\mathrm{ult}}/\left(\mathrm{AD}s_{u}\right)$ is the moment capacity. For embedded foundations, the torsional capacity is related only to the foundation base interface (side and top interfaces are smooth), therefore, the embedment ratio does not affect the torsional capacity.

**Figure 8.**
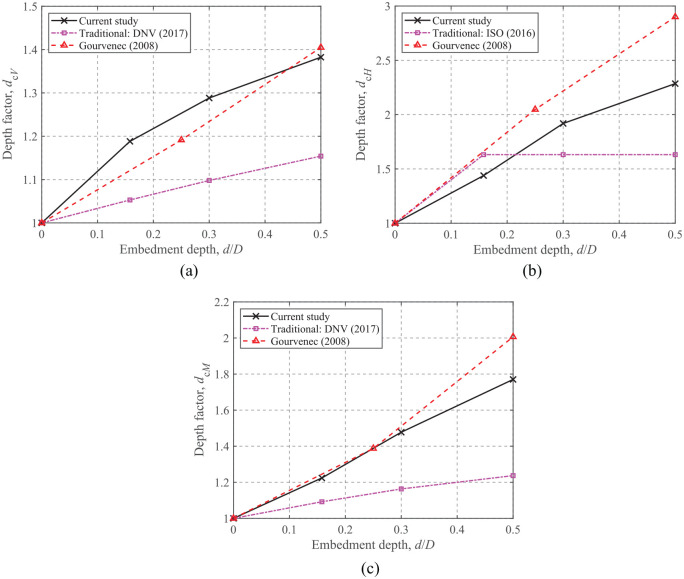
Depth correction factors of uniaxial capacities for embedded foundations: (a) *V*_ult_, (b) *H*_ult_, and (c) *M*_ult_.

The relationships between the depth factors and embedment depth ratios are shown in Figure 8 along with results from design standards and previous results. As DNV GL (2016) does not provide embedment factors for traditional methods, the embedment factors recommended by DNV GL (2017) and ISO (2016) are applied. The FE results of Gourvenec (2008) for embedded strip foundations with unlimited-tension interfaces are also compared in Figure 8. Compared to the current FE results, DNV GL (2017) provides more conservative results with differences of about 22% and 47% for the vertical and moment depth factors, respectively. Comparable results can be seen for the vertical capacity, while the current depth factors for horizontal and moment capacities are smaller than those of Gourvenec (2008) due to the assumption of unlimited-tension interfaces. Since DNV GL (2017) does not consider the embedment effect for horizontal capacity, ISO (2016) is used for comparison. The total horizontal capacity given by ISO (2016) is equal to the base friction (i.e. $A_{\mathrm{base}}\times s_{u}$ ) plus the additional side resistance due to the difference between the active and passive resistances. Therefore, the horizontal capacity estimated by ISO (2016) remains constant when the foundation is fully embedded in the soil, as shown in Figure 8(b). Thus, this calculation is more conservative for embedded foundations. However, due to the possible side gaps occurring between the foundation and soil caused by installation disturbance and possible cyclic loading processes, this approach appears to be reasonable in practice.

### Horizontal-Vertical loading

**Figure 9.**
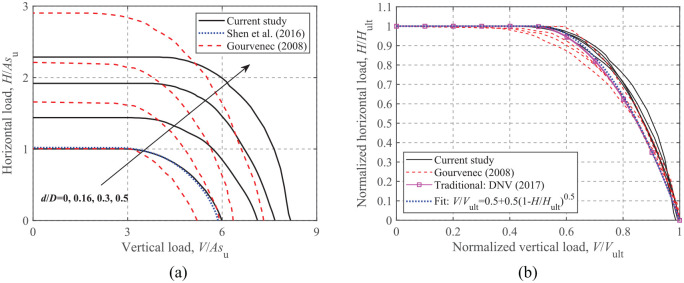
*H*-*V* failure envelopes: (a) dimensionless and (b) normalized.

Figure 9 shows the *H-V* envelopes for the different embedment ratios. The dimensionless loads shown in Figure 9(a) represent the absolute size of the envelopes, and the normalized envelopes shown in Figure 9(b) are more appropriate for developing analytical equations.

As shown in Figure 9(a), the failure envelope expands with increasing embedment ratio. Compared to the failure envelopes for strip foundations with an unlimited-tension interface developed by Gourvenec (2008), circular foundations with a zero-tension interface have larger vertical capacities, but smaller horizontal capacities due to the friction on the foundation top surface and the developed tension stresses on the foundation side for an unlimited-tension interface. Figure 9(b) shows that the failure envelopes normalized by their corresponding ultimate capacities fall into a tighter band than those of Gourvenec (2008), whilst the DNV GL (2017) approach lies slightly inside the current envelopes. A curve fit using Green’s solution, which is widely used to characterize the *H-V* envelopes (see equation (10)), also provides a satisfactory comparison with the current FE results, as shown in Figure 9(b).

(10) $$V/V_{\mathrm{ult}}=0.5+0.5\sqrt{1-H/H_{\mathrm{ult}}}$$

### Moment-Vertical loading

**Figure 10.**
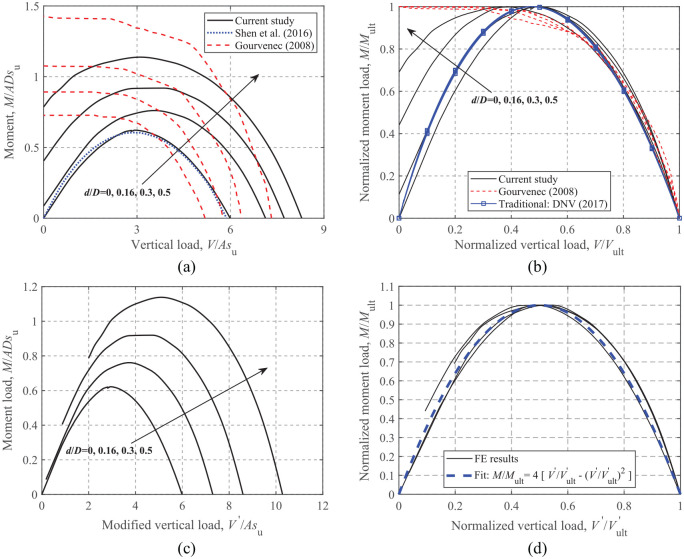
*M*-*V* failure envelopes: (a) dimensionless, (b) normalized, (c) dimensionless, modified, and (d) normalized, modified.

Figure 10(a) and (b) show the dimensionless and normalized *M-V* envelopes for embedded foundations (*H *= 0). However, the current study shows different patterns for the *M-V* envelopes compared to those of Gourvenec (2008). This is because an unlimited-tension interface can result in consistently increasing moment capacity with the decrease of vertical load, while a reduction of moment occurs for a zero-tension interface owing to the separation of foundation from the soil under relatively small vertical loads. Figure 10(b) shows that DNV GL (2017) only provides *M-V* curves that resemble the current envelopes for a surface foundation irrespective of embedment ratios. In contrast, the FE results show that embedded foundations can sustain increasing moments with depth at zero vertical load, because the side and top soils (i.e. soils above the foundation base) provide additional resistance even in the absence of vertical loads. As shown Figure 10(b), due to the non-zero intercepts with the moment axis, the fitted equation for a circular surface foundation with a zero-tension interface (see equation (11)) cannot be directly extended to the embedded cases. Therefore, a more generalized form of equation should be developed to account for the embedment effect.

(11) $$M/M_{\mathrm{ult}}=4\left[V/V_{\mathrm{ult}}-{\left(V/V_{\mathrm{ult}}\right)}^{2}\right]$$

Figure 10(b) shows that the *M-V* failure envelopes for embedded foundations still follow parabolic forms, although they are not complete curves. To transform these incomplete envelopes into the same form to create a complete surface that passes through the origin, the envelopes shown in Figure 10(a) are shifted to the right along the *x* axis, which is equivalent to:

(12) $$V^{\prime }=V+\Delta V\;\mathrm{and}\;V_{\mathrm{ult}}^{\prime }=V_{\mathrm{ult}}+\Delta V$$

where ΔV represents the amount of offset and can be defined as $\Delta V=V_{\mathrm{ult}}\Delta f\left(d/D\right)$ , where f(d/D) is a function of the embedment ratio. Curve fitting shows that $f\left(d/D\right)=0.74{\left(d/D\right)}^{2}+0.12d/D$ provides a satisfactory prediction. New normalized failure envelopes (i.e. $M/M_{\mathrm{ult}}\sim V^{\prime }/V_{\mathrm{ult}}^{\prime }$ ) are then obtained based on the modified failure envelopes, as shown in Figure 10(d). The figure also shows that equation (11) can still be used to model the modified curves.

### Torsion-Vertical loading

**Figure 11.**
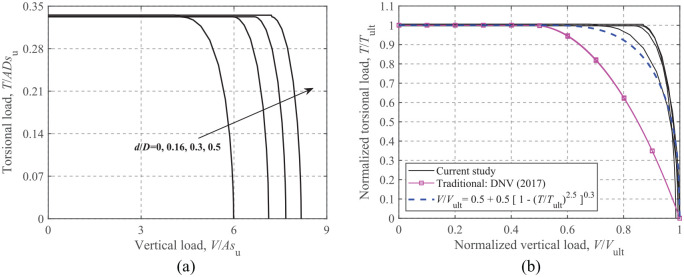
*T*-*V* failure envelopes: (a) dimensionless and (b) normalized.

Figure 11 shows the dimensionless and normalized *T-V* failure envelopes for embedded circular foundations. Similar shapes for the failure envelopes can be observed from Figure 11(a). Normalized failure envelopes in Figure 11(b) show that DNV GL (2017) significantly underestimates the torsional bearing capacity when*V*/*V*_ult_ > 0.50. The analytical equation (see equation (4)) for a circular foundation with an unlimited-tension interface proposed by Abyaneh et al. (2015) is also compared in Figure 11(b). Although slight discrepancies between the equation and FE results are observed, this equation can still provide reasonable and conservative predictions regardless of the embedment ratio.

### Moment-Horizontal loading

**Figure 12.**
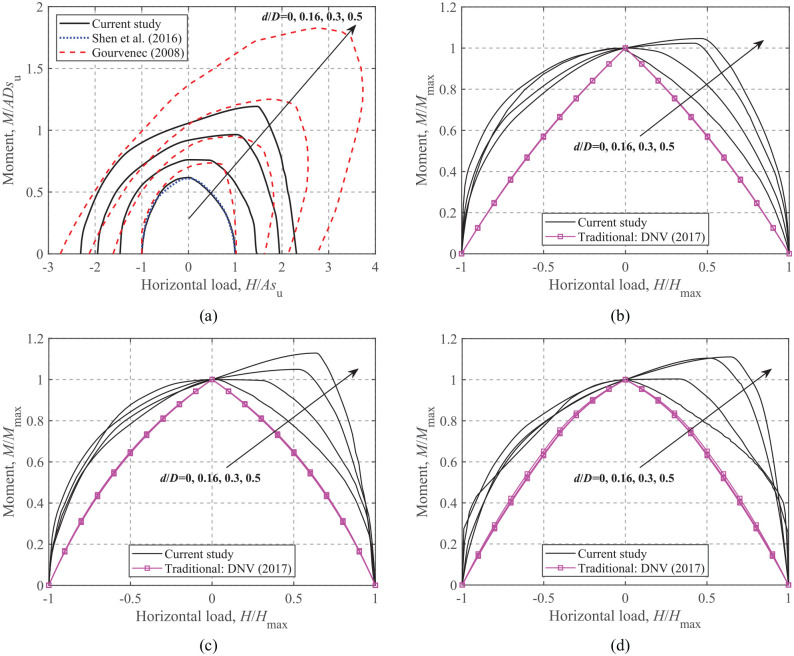
*M*-*H* failure envelopes: (a) dimensionless, *V*/*V*_ult_ = 0.50, (b) normalized, *V*/*V*_ult_ = 0.75, (c) normalized, *V*/*V*_ult_ = 0.50, and (d) normalized, *V*/*V*_ult_ = 0.25.

Figure 12 shows the dimensionless and normalized *M-H* failure envelopes at *V*/*V*_ult_ = 0.25, 0.50, and 0.75 for embedded foundations. Only dimensionless envelopes at *V*/*V*_ult_ = 0.50 are presented (see Figure 12(a)) to show the evolution of the absolute size of the envelope. As shown in Figure 12(a), the failure envelops for a strip foundation with an unlimited-tension interface obtained by Gourvenec (2008) are consistently larger than the current FE results due to different foundation geometries and interface conditions. It should also be noted that the failure envelope for a surface foundation is almost symmetric about *H *= 0, however, the foundation embedment gradually increases the degree of asymmetry, indicating that the *M-H* capacity in the (+*M*, +*H*) region is larger than that in the (+*M*, −*H*) region. This is due to the cross-coupling effect between horizontal loads and moments.

**Figure 13.**
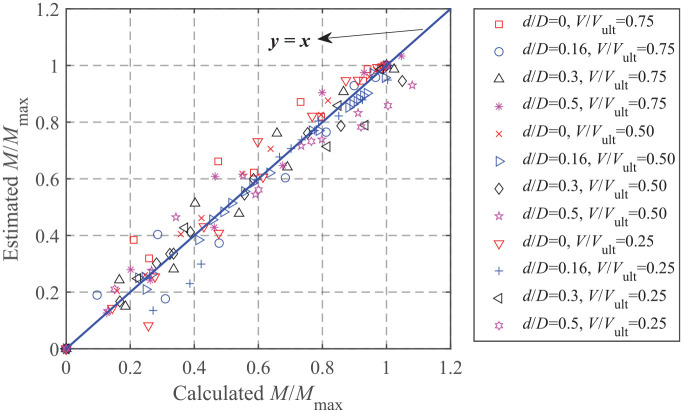
Fitting of the probe values of *M*-*H* failure envelopes.

Figure 12(b) to (d) shows the *M-H* failure envelopes normalized by the corresponding maximum horizontal load and moment (i.e. intersections of the failure envelopes with the horizontal load and moment axes) along with the results of DNV GL (2017) The failure envelopes given by DNV GL (2017) are more conservative and symmetric about the moment axis regardless of embedment ratios, indicating no coupling effects considered. The form of equation for *M-H* failure envelopes accounting for the effect of foundation embedment is expressed as:

(13) $${\left(H/H_{\max}\right)}^{2}+{\left(M/M_{\max}\right)}^{2}\left[1-h\left(H/H_{\max}\right)\right]=1$$

where *h* is a function of *d*/*D*, that is, h(d/D)=1.46(d/D)−0.14 . The comparison between the calculated and estimated $M/M_{\max}$ is shown in Figure 13 and shows a good fit.

### Horizontal-Torsional loading

**Figure 14.**
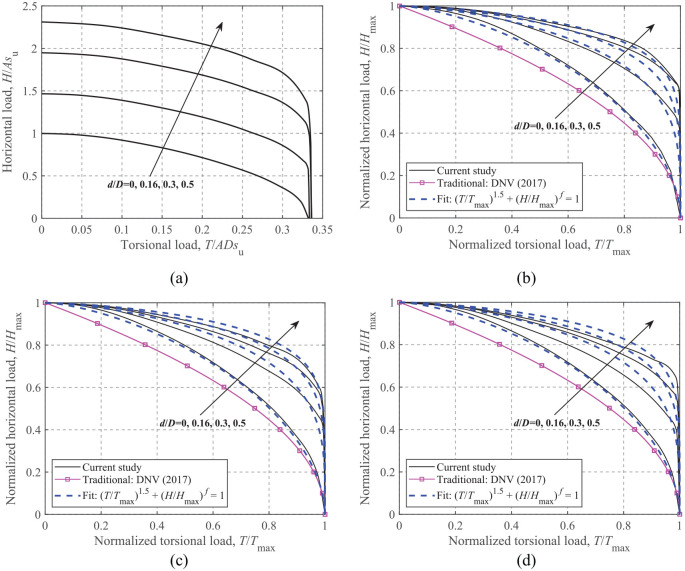
*H*-*T* failure envelopes: (a) dimensionless, *V*/*V*_ult_ = 0.50, (b) normalized, *V*/*V*_ult_ = 0.75, (c) normalized, *V*/*V*_ult_ = 0.50, and (d) normalized, *V*/*V*_ult_ = 0.25.

Figure 14 shows the dimensionless and normalized failure envelopes under horizontal and torsional loads (zero moment). Figure 14a shows the expansion of the absolute size of the *H-T* failure envelopes with the embedment ratio. To describe these curves using a unique expression irrespective of vertical load levels and embedment ratios, the corresponding maximum horizontal and torsional loads are adopted for normalization, as shown in Figure 14(b) to (d). The comparison shows that the traditional approach consistently leads to more conservative *H-T* failure envelopes, and the embedment effect has no influence on the traditional *H-T* failure envelope. In contrast, the FE results show that foundation embedment significantly affects the *H-T* failure envelope even after normalization, but this effect decreases gradually with the embedment ratio.

Due to the dispersion of the normalized curves caused by foundation embedment, the embedment effect needs to be considered in developing analytical expressions. The general form of formula for the *H-T* failure envelopes is taken as:

(14) $${\left(T/T_{\max}\right)}^{1.5}+{\left(H/H_{\max}\right)}^{f}=1$$

where *f* is a function of the embedment ratio. Curve-fitting shows that $f\left(d/D\right)=-7.74{\left(d/D\right)}^{2}+13.5d/D+1.83$ is a good approximation. The analytical curves of equation (14) are also compared in Figure 14, where reasonable predictions are observed apart from slight overestimations for embedded foundations at *V*/*V*_ult_ = 0.25.

### Moment-Torsional loading

**Figure 15.**
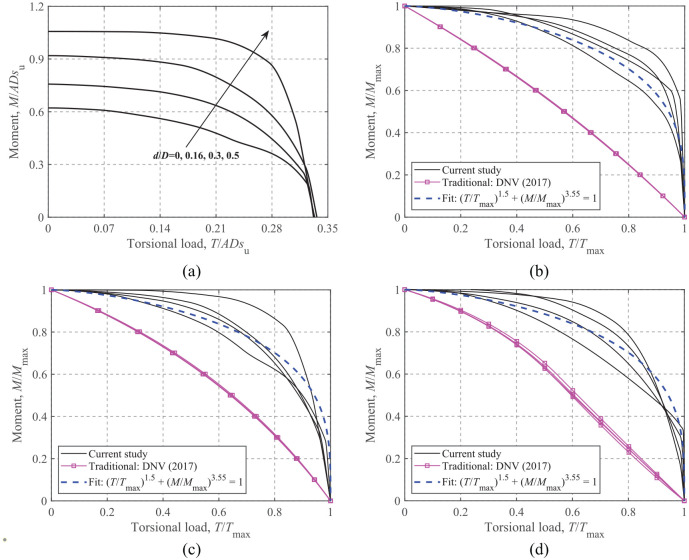
*M*-*T* failure envelopes: (a) dimensionless, *V*/*V*_ult_ = 0.50, (b) normalized, *V*/*V*_ult_ = 0.75, (c) normalized, *V*/*V*_ult_ = 0.50, and (d) normalized, *V*/*V*_ult_ = 0.25.

The dimensionless and normalized failure envelopes for *M-T* loading are shown Figure 15. Similar to the *H-T* envelopes, a significant expansion of the curve size with the embedment ratio is seen from Figure 15(a). The failure envelopes obtained by the conventional approach (DNV GL, 2017) lie significantly inside the current results, as shown in Figure 15(b) to (d). Foundation embedment also affects the normalized *M-T* failure envelope; however, unlike the *H-T* envelopes, the *M-T* failure envelopes for different embedment ratios blend together, and no consistent trend is observed. This feature does not easily lend itself to any simple form of expression that can account for the embedment effect. As a first approximation, a unique equation, which follows the overall trend of the envelopes, is recommended:

(15) $${\left(T/T_{\max}\right)}^{1.5}+{\left(M/M_{\max}\right)}^{3.55}=1$$

As shown in Figure 15, this expression provides reasonable fits, although it is slightly unconservative for surface foundations when *V*/*V*_ult _> 0.40.

## Full 4-D failure envelope in VHMT loading space

This section derives the analytical expression for the full 4-D VHMT failure envelope. Three sets of notations are defined: (1) $V_{\mathrm{ult}}$ , $H_{\mathrm{ult}}$ , $M_{\mathrm{ult}}$ , $T_{\mathrm{ult}}$ —uniaxial ultimate capacity; (2) $H_{\max}$ , $M_{\max}$ , $T_{\max}$ —maximum capacity at a given level of vertical load without other load components; (3) $H_{\max}^{\prime }$ , $M_{\max}^{\prime }$ —reduced maximum capacity at a given level of vertical load with *T*≠0.

Based on the above notations and the equations used in the previous sections, the general forms of all of the equations are summarized in equations (16)∼ (18). Specific expressions for different soil and foundation conditions can be found in the previous sections.

(16) $$\frac{H_{\max}}{H_{\mathrm{ult}}}=f_{h}\left(\frac{V}{V_{\mathrm{ult}}}\right),\;\frac{M_{\max}}{M_{\mathrm{ult}}}=f_{m}\left(\frac{V}{V_{\mathrm{ult}}}\right),\;\frac{T_{\max}}{T_{\mathrm{ult}}}=f_{t}\left(\frac{V}{V_{\mathrm{ult}}}\right)$$

(17) $$f_{\mathrm{mh}}\left(\frac{H}{H_{\max}},\frac{M}{M_{\max}}\;\right)=1$$

(18) $${\left(\frac{H_{\max}^{\prime }}{H_{\max}}\right)}^{c}+{\left(\frac{T}{T_{\max}}\right)}^{d}=1,\;{\left(\frac{M_{\max}^{\prime }}{M_{\max}}\right)}^{e}+{\left(\frac{T}{T_{\max}}\right)}^{f}=1$$

**Figure 16.**
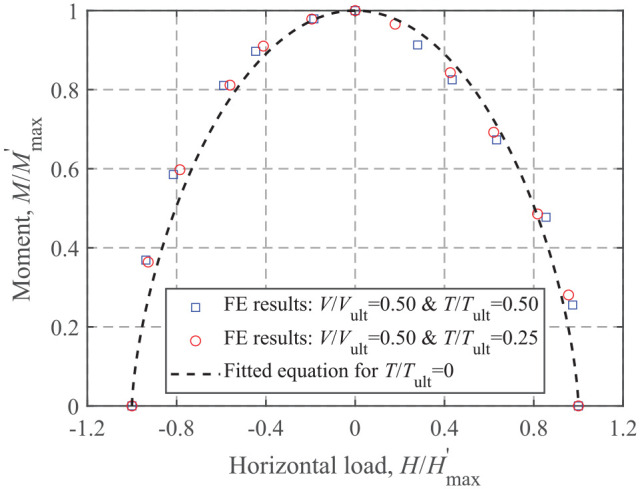
*M-H* envelope for a circular surface foundation on a homogeneous soil at *V*/*V*_ult_ = 0.50 for *T*≠0.

Equation (17) for the *M-H* failure envelope is taken as the basic function. Due to the very similar shape of the *M-H* failure envelope (only the sizes are different), it is reasonable to assume that equation (17) is still applicable for the *M-H* failure envelope under the condition of *T*≠0 when normalized by the corresponding maximum values, $H_{\max}^{\prime }$ and $M_{\max}^{\prime }$ (these reduce to $H_{\max}$ and $M_{\max}$ in equation (17) if *T *= 0). An example of the *M-H* envelope for a non-zero torsional load is shown in Figure 16, indicating a good fit. Therefore, equation (17) is replaced by a more generalized form:

(19) $$f_{\mathrm{mh}}\left(\frac{H}{H_{\max}^{\prime }},\frac{M}{M_{\max}^{\prime }}\;\right)=1$$

Mathematical manipulations allow the formulation of an analytical 4-D VHMT expression in terms of $V/V_{\mathrm{ult}}$ , $H/H_{\mathrm{ult}}$ , $M/M_{\mathrm{ult}}$ , and $T/T_{\mathrm{ult}}$ :

(20) $$f_{\mathrm{mh}}\left(\frac{H/H_{\mathrm{ult}}}{{\left[1-{\left(\frac{T/T_{\mathrm{ult}}}{f_{t}\left(V/V_{\mathrm{ult}}\right)}\right)}^{d}\right]}^{\frac{1}{c}}\;\cdot \;f_{h}\left(V/V_{\mathrm{ult}}\right)},\;\frac{M/M_{\mathrm{ult}}}{{\left[1-{\left(\frac{T/T_{\mathrm{ult}}}{f_{t}\left(V/V_{\mathrm{ult}}\right)}\right)}^{f}\right]}^{\frac{1}{e}}\;\cdot \;f_{m}\left(V/V_{\mathrm{ult}}\right)}\;\right)=1$$

In design practice, factored design loads, VHMT, can be directly substituted into the left-hand side of equation (20); values less than 1 represent a sufficient ultimate limit design and vice versa. For embedded foundations, it should be noted that the design vertical load, *V*, should be reduced by γdA to account for the additional surcharge caused by the soil above the foundation base.

**Figure 17.**
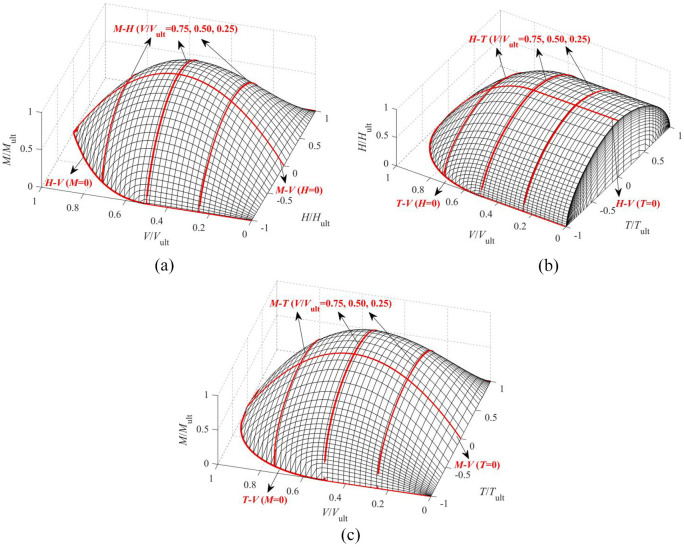
3-D failure surfaces for a circular surface foundation on non-homogeneous soils: (a) VHM at *T *= 0, (b) VHT at *M *= 0, and (c) VMT at *H *= 0.

As an example, the full 4-D VHMT failure envelope for a surface foundation on non-homogeneous soils is shown. The equations for *H-V, M-V* and *M-H* failure envelopes have been given by Shen et al. (2016) and the expressions for *T-V, H-T*, and *M-T* failure envelopes can be found in the previous sections. To visualize the shape of the full 4-D failure surface, three special 3-D failure surfaces in terms of $V/V_{\mathrm{ult}}$ , $H/H_{\mathrm{ult}}$ , $M/M_{\mathrm{ult}}$ , and $T/T_{\mathrm{ult}}$ (i.e. VHM failure surface at *T *= 0, VHT failure surface at *M *= 0 and VMT failure surface at *H *= 0) are presented in Figure 17. The specific curves derived from FE analyses are also shown for comparison. The shape of the 3-D VHM failure surface (at *T *= 0) is similar to that obtained by Taiebat and Carter (2010) using a semi-analytical FE approach. For the VHT and VMT failure surfaces, the portion of *T *< 0 is also incorporated considering the symmetry about the plane of *T *= 0.

## Conclusions

The general VHMT failure envelopes of circular foundations for onshore wind turbines with a zero-tension interface for undrained soils have been studied using FE analyses. For surface foundations on a non-homogeneous soil, the analytical *V-T* and *H-T* and *M-T* failure envelopes have been provided considering four soil strength heterogeneity ratios. The results indicate that the torsional bearing capacity factor is equal to 1/3, and torsional loads reduce the VHM capacity of circular foundations. Embedded foundations in a homogeneous soil with four embedment depths were taken into consideration. The effect of foundation embedment on the VHMT failure envelope was studied, and analytical formulas have been proposed. As expected, foundation embedment significantly increases the capacity of circular foundations. More specifically, for an embedment ratio of 0.5, compared to surface foundations, the uniaxial vertical, horizontal and moment capacities increase by about 38%, 130%, and 78%, respectively. In addition, compared to the FE results, DNV GL (2017) provides more conservative results, for example, about 22% and 47% lower vertical and moment depth factors than the current results. To facilitate the design application of the failure envelope method, a full 4-D analytical expression for the VHMT failure envelope was derived based on the calculated VHMT failure envelopes. These approaches should aid the assessment of the ultimate limit states of wind turbine foundations under complex VHMT loading conditions. Since the failure envelopes are affected by the soil and foundation conditions (e.g. foundation shapes, soil layers and interface conditions), a series of 4-D failure envelope equations for common soil and foundation conditions can be derived following the same procedures presented in this paper for future research.
